# Suicide attempts during pregnancy in South Africa

**DOI:** 10.4102/sajpsychiatry.v24i0.1154

**Published:** 2018-04-16

**Authors:** Naseema B.M. Vawda

**Affiliations:** 1 Department of Behavioural Medicine, University of KwaZulu-Natal, South Africa

## Abstract

**Background:**

Research on suicide attempts during pregnancy is limited as these are considered to be rare in the perinatal period.

**Aim:**

A retrospective pilot study was undertaken to establish what percentage of suicide attempters admitted to a hospital were pregnant and to identify their clinical and sociodemographic characteristics.

**Setting:**

The study was conducted at a tertiary hospital in Durban, South Africa.

**Methods:**

A retrospective chart review was undertaken of all female patients admitted following suicide attempts over a period of 1 year. Clinical and sociodemographic data of pregnant suicide attempters were extracted.

**Results:**

Of 27 charts reviewed, 33% (*n* = 9) patients were pregnant at the time of the attempt. V Code diagnoses predominated, followed by major depressive disorder. Past psychiatric diagnoses and suicide attempts were also present

**Conclusion:**

Suicide attempts during pregnancy are not rare. Pregnant women should be routinely screened for prior suicide attempts, depression and stressors as part of perinatal assessments.

## Background

Research on suicidal behaviour indicates that males are four times more likely to commit suicide than females, but that women are more likely to have suicidal thoughts.^1^ Maternal and child health issues together with injury and violence have been identified as some of the quadruple burdens of disease facing the South African health care system.^2^ However, research on self-injury such as suicide attempts in pregnancy is limited owing to a belief that pregnancy is protective against suicide and suicidal behaviour.^3^ One of the few studies which examined suicide attempts in pregnancy in a developing country found a rate of 5.0%.^4^

Given that the suicide rate for females in South Africa is 4.5 per 100 000,^5^ with twice as many women reporting suicide attempts as men (3.8% vs. 1.8%)^6^ and that pregnancy is a critical time period for most women, this area is under-researched in South Africa.

## Aim

This pilot study was undertaken to establish what percentage of females admitted following a suicide attempt to a government tertiary hospital were pregnant and to establish associated clinical and sociodemographic factors

## Method

A retrospective review of medical and psychological charts of all female patients admitted to a tertiary hospital in Durban following a suicide attempt over one randomly chosen year (01 January to 31 December 2014) was conducted. The policy of the hospital, as established by the clinical psychology department with all referring departments, is that all patients presenting with suicidal behaviour (including attempts) must be admitted, assessed and managed by clinical psychologists within 24–48 hours of admission, once medically stable and prior to discharge.

The clinical and sociodemographic data of pregnant suicide attempters was extracted from their charts, after ethical approval was obtained from the University of KwaZulu-Natal (BREC BE 264/16).The Diagnostic and Statistical Manual of Mental Disorders 5 (DSM 5) was used for diagnoses.

## Results

Of a total of 27 female patients, all of black ethnicity, admitted following a suicide attempt, 33.3% (*n* = 9) were pregnant at the time of the attempt. The characteristics of these nine patients are presented in Table 1.

**TABLE 1:** Clinical and sociodemographic characteristics.

Clinical and Sociodemographic characteristics	*N*	%
**Referred from**		
Medical wards	5	55.6
Obstetric and gynaecology wards	4	44.4
**Living arrangements**		
with partner	3	33.3
with family of origin	4	44.5
with extended family	1	11.1
on own	1	11.1
**Educational level**		
Tertiary	3	33.3
Grade 12	4	44.5
Grade 11	1	11.1
Grade 9	1	11.1
**Method**		
over-the-counter medications	6	66. 7
own anti-retroviral medication (ARM)	1	11.1
spirits	1	11.1
combination (own ARM and petrol)	1	11.1
**Medical history**		
HIV-positive	4	44.5
HIV unknown/undocumented	5	55.5
**Precipitants of attempts**		
partner relationship problems	5	55.6
family relationship problems	2	22.2
witnessing murder of significant other	1	11.1
financial problems	1	11.1
Past psychiatric history	2	22.2
Previous suicide attempts	2	22.2
**Psychiatric diagnoses (current)**		
Major depressive disorder	3	33.3
V Code	5	55.6
Acute stress disorder	1	11.1
**Management (combined)**		
psychotherapy and psychotropic medication	4	44.4
psychotherapy and social work	5	55.6

ARM, anti-retroviral medication; HIV, human immunodeficiency virus.

The mean age was 23.4 years (range 15 to 33; s.d. 5.93). The mean foetal age at the time of the attempt was 22.67 weeks (range: 7 – 38 weeks). The majority were referred from the medical wards, having overdosed on various over- the-counter and prescribed medications (ferrous sulphate, paracetamol and anti-retrovirals). V Codes constituted the majority of the diagnoses. Interpersonal conflicts with partners over issues of infidelity or denial of paternity and conflicts with the family or caregivers around their disapproval of the pregnancy were key precipitants. Major depressive disorder (MDD) was diagnosed in 33% of the patients.

A previous diagnosis of MDD was reported by 22.2%, who were also diagnosed as MDD following the current attempt, but they were not on antidepressants at the time of attempt. Twenty-two per cent of the patients reported one previous attempt each for which no psychological/psychiatric intervention was sought. All patients received psychotherapy, while 44.0% were also referred to social workers and 55.0% to the psychiatry department where psychotropic medication was prescribed.

## Discussion

One-third of all female patients admitted following a suicide attempt were pregnant, indicating that pregnancy is not a protective factor for suicide attempts in this sample.

This study supports research indicating that relational conflicts are key stressors or precipitants.^7^ While studies in developed countries found that intimate partner violence was a precipitant for either major depression or suicide attempts and that spousal physical abuse and poor relationships with mothers-in-law were stressors in developing countries,^4,8^ such factors have not been found to be precipitants in this study.

That one-third of the sample was diagnosed with MDD is in keeping with data reported in developing countries.^7,8^ While participants diagnosed as having an MDD had also made previous suicide attempts while pregnant, no mental health help had been sought. This indicates missed opportunities for intervention given that a prior history of suicide attempts and depressive symptoms as risk factors for future suicide attempts are well documented.^9^

Maternal perinatal depression can have adverse outcomes for both mother and child health such as: impaired feto-placental functioning, low foetal growth, pre-eclampsia, premature delivery, low birthweight, and negative developmental and psychological outcomes for the child,^7^ such as poor maternal-child attachment, child abuse or neglect.

While only one suicide attempt was owing to trauma exposure, given that more than 75% of South Africans report exposure to lifetime trauma and witnessing violence is a significant predictor of lifetime suicide attempts,^10^ its role as a precipitant of suicide attempts during pregnancy needs to be further investigated.

The HIV status of more than half the cases was unknown. This indicates the need for better record keeping and missed opportunities for voluntary counselling and testing as entry points into intervention for suicidal behaviour. During times of crisis or stress, cognitive deficits resulting from HIV such as problems with cognitive flexibility, concentration and memory may overwhelm individuals, resulting in impulsive suicide attempts.^11^

Some limitations of this study are: the small sample size, since the data was gathered on one ethnic group only (as there were no other ethnic groups admitted during the study period). This limits generalisability to other communities and populations. However, as only patients with severe complications are admitted to tertiary level hospitals, these findings may be an under-representation of the problem. Pregnant suicide attempters may have been admitted to other hospitals, may have been seen by general practitioners or may not have sought or been taken for medical treatment.

## Conclusion

This pilot study adds to the body of literature on suicidal behaviour and maternal mental health in South Africa. Although the sample size is small, the findings indicate that suicide attempts are not rare in pregnant women and that pregnancy may not be a protective factor against suicide attempts. Studies need to be undertaken with larger samples, different ethnicities and various socioeconomic groups. Medical practitioners should routinely monitor pregnant patients for stressors, mental illness and previous suicide attempts. When admitted, pregnant suicide attempters should be co-managed holistically by obstetricians, physicians and mental health professionals throughout pregnancy and antenatally. The goal should be the early identification of stressors and the prevention of suicide attempts, thus ensuring viable foetal outcomes and the prevention of negative long-term outcomes, such as child abuse.
